# American Society of Dental Surgeons

**Published:** 1852-10

**Authors:** 


### EDITORIAL DEPARTMENT.

*American Society of Dental Surgeons.*—The thirteenth annual meeting of the American Society of Dental Surgeons, was held at the Ocean House, Newport, R. I. We learn from the minutes, loaned us by the Secretary, that the following business was transacted.

At 10 o'clock, Tuesday morning, August 3d, the meeting was called to order by the President, Dr. E. Parmly of New York. Eighteen members as well as several other gentlemen of the profession were present. The minutes of the preceding meeting having been read and adopted, an invitation, on motion of Professor E. Townsend, was given to members of the profession of Newport and others, to attend the sessions of the Society.

The order of the business for the present meeting was then announced by the Chairman of the Executive Council. After which, the Secretary reported his correspondence since the preceding meeting. The Treasurer next made his report, whose accounts were referred to a committee, composed of Drs. J. D. White, and J. H. Foster to audit.

The resolution offered by Dr. E. Townsend at the preceding meeting in relation to the admission of delegates from dental societies and colleges, coming up for action, was laid over until the next meeting.

The committee appointed to revise the constitution, reported progress and were continued.

The committee appointed to audit the Treasurer's accounts, reported that they had found them to be correct, which report was accepted and the committee discharged.

Dr. E. Townsend, committee on practical dentistry, was, after some remarks, on motion of Dr. Hill, discharged from further duty.

A series of resolutions were offered by Dr. Townsend, on the subject of dental colleges, which, after some discussion, were referred to a committee, consisting of Drs. J. H. Foster, A. Robertson, A. Hill, E. G. Tucker, and A. C. Hawes.

The Society now adjourned to 9, A. M. Wednesday morning.

Wednesday, 9, A. M. The Society met agreeably to adjournment, and the minutes of the preceding day were read and approved. After which, the chairman of the committee on the causes and treatment of irregularity of the teeth, offered some remarks explanatory of the delay of the committee to report. The subject elicited some debate, and the committee, on motion of Dr. Dunning, were discharged.

Dr. S. P. Hullihen offered the following resolution:

"*Resolved,* That this association offer an award of a gold medal of the value of twenty-five dollars for the best paper on the causes and correction of dental irregularities, to be adjudged by a special committee to be appointed at the next annual meeting to examine the merits of all papers offered in competition for the prize. All papers offered the society in competition shall become the property of the association."

The above resolution was adopted.

The committee on Dr. Townsend's resolutions on the subject of dental colleges and dental education, offered the following resolutions:

1. "*Resolved,* That in the judgment of this convention, our profession has now fully reached that stage of progress at which provision for thorough collegiate instruction for students in dental surgery has become necessary, obligatory, and practicable, and that the establishment of *dental* colleges at the centre points in the United States, is demanded by our relations and responsibilities to the public and by the interest and honor of our science.

2. "*Resolved,* That we are clear in the opinion that all the departments of medical and surgical learning properly involved in our branch of the healing art, are not only capable of tuition by the collegiate method, but as much, or more than any other specialty of medical science require the apparatus of such institutions for the successful training of its students.

3. "*Resolved,* That the addition of a single chair of dentistry to our medical colleges while it would supply to their students such generalities of dental theory as a complete medical education *requires,* would be inadequate for the *education* of dentists proper, such a system being incapable of the required accommodation in its several chairs of anatomy, surgery materia medica, and practice to the special necessities of dentistry, and having no room for its peculiar departments of mechanical and operative instructions, and therefore its incapability to afford clinical practice, and the experience of the laboratory under skillful direction or the time necessary to make any of them available.

4. "*Resolved* That in the judgment of this society, the appointment of a dental lecturer or professorship in each of our medical colleges would result in many advantages to the students and practitioners of general medicine and surgery as well as to the community at large, affording them much useful knowledge which has not hitherto been taught in those schools, and consequent facilities in the practice of certain branches of medical science.

All of which is respectfully submitted.

Signed,

J. H. FOSTER,<br>

A. ROBERTSON,<br>

A. HILL,<br>

E. G. TUCKER,<br>

A. C. HAWES, } *Committee.*

The foregoing report was accepted and the committee discharged.

The resolutions after some discussion were laid on the table.

Dr. J. D. White read a paper on the anatomy and physiology of dentine.

Dr. E. Townsend read a paper on professional fees, which will be found in another part of the present number of the Journal.

Dr. E. G. Tucker read a paper on the use of gum elastic rings in the treatment of irregularity of the teeth. This will be found in another part of the present number of the Journal.

Dr. A. Robertson read a paper on the morbid effects of diseased teeth, which we publish in the present number of the Journal.

The thanks of the society were offered the above named gentlemen, and copies of their essays requested for publication, in the dental journals.

The committee appointed at the preceding annual meeting to revise the constitution of the society, were, on motion of Dr. Hullihen, discharged.

Dr. J. D. White then offered the following resolution :

"*Resolved,* That a committee of five be appointed to draft a new constitution and by-laws for the government of the society ;" which having been adopted, the following gentlemen were nominated and appointed on the committee, viz. Drs. S. P. Hullihen, J. D. White, J. H. Foster, E. J. Dunning and E. Parmly.

The officers of last year were re-elected for the year ensuing.

Dr. Hullihen was appointed to deliver the anniversary address in 1853. Drs. E. J. Dunning, E. Townsend, B. Lord, M. K. Bridges, and J. H. Foster, were appointed to prepare dissertations on subjects connected with dental theory or practice, to be read at the next meeting.

The thanks of the society were tendered to Messrs. Jones, White and McCurdy for the faithful manner in which they had the proceedings of the meeting of the previous year reported.

During the afternoon session, Dr. Cone read a paper on Dr. Hullihen's method of filling teeth after the dental pulp has become exposed, embodying a description given by Dr. H., of his method of procedure, and for which the thanks of the society were tendered Drs. H. and C., with a request that a copy be furnished for publication.

The Society having completed its business, adjourned to meet at West Point, New York, second Tuesday of August, 1853.

